# Anatase-Wrapped Rutile Nanorods as an Effective Electron Collector in Hybrid Photoanodes for Visible Light-Driven Oxygen Evolution

**DOI:** 10.3389/fchem.2021.709903

**Published:** 2021-08-18

**Authors:** Ruihao Gong, Dariusz Mitoraj, Robert Leiter, Manuel Mundszinger, Alexander K. Mengele, Igor Krivtsov, Johannes Biskupek, Ute Kaiser, Radim Beranek, Sven Rau

**Affiliations:** ^1^ Institute for Inorganic Chemistry I, Ulm University, Ulm, Germany; ^2^ Institute of Electrochemistry, Ulm University, Ulm, Germany; ^3^ Electron Microscopy Group of Materials Science, Ulm University, Ulm, Germany

**Keywords:** anatase-wrapped rutile nanorods, electron collector, hybrid, photoanode, visible light, oxygen evolution

## Abstract

Arrays of single crystal TiO_2_ rutile nanorods (RNRs) appear highly promising as electron-collecting substrates in hybrid photoanodes as the RNRs offer direct charge carriers transport pathways, contrary to the conventional electrodes prepared from TiO_2_ powders that suffer from the numerous charge traps at the grain boundaries. However, the specific surface area of the nanorods is highly limited by their smooth morphology, which might be detrimental in view of utilizing the RNR as a substrate for immobilizing other functional materials. In this study, we developed a novel anatase-wrapped RNR (ARNR) material fabricated by a facile seed layer-free hydrothermal method. The ARNR comprises polycrystalline anatase nanoparticles formed on the surface of RNR, resulting in a large surface area that provides more deposition sites compared to the bare nanorods. Herein, we functionalize ARNR and RNR electrodes with polymeric carbon nitride (CN_x_) coupled with a CoO(OH)_x_ cocatalyst for dioxygen evolution. The anatase wrapping of the rutile nanorod scaffold is found to be crucial for effective deposition of CN_x_ and for improved photoanode operation in visible light-driven (*λ* > 420 nm) oxygen evolution, yielding a significant enhancement of photocurrent (by the factor of ∼3.7 at 1.23 V vs*.* RHE) and faradaic efficiency of oxygen evolution (by the factor of ∼2) as compared to photoanodes without anatase interlayer. This study thus highlights the importance of careful interfacial engineering in constructing photoelectrocatalytic systems for solar energy conversion and paves the way for the use of ARNR-based electron collectors in further hybrid and composite photochemical architectures for solar fuel production.

## Introduction

Hydrogen is considered a promising energy carrier that can be generated by splitting water into H_2_ and O_2_ using renewable energy sources. Apart from other technological concepts, solar-driven water splitting in photoelectrochemical (PEC) cells is considered a viable approach to directly convert and store solar energy in the form of chemical energy either directly in hydrogen or in high-energy compounds (e.g., hydrocarbons or alcohols) that can be subsequently obtained by reduction of CO_2_ with hydrogen (Giménez and Bisquert, 2016; Telley et al., 2018). In contrast to kinetically relatively easy H_2_ evolution reaction (HER), O_2_ evolution reaction (OER) is the typical rate-limiting bottleneck of all water-splitting devices due to the complex four-electron transfer process and the slower kinetics (Deng and Tüysüz, 2014). Therefore, the development of efficient photoanodes for PEC water splitting is of high importance. Most investigated photoanodes for PEC cells are based either on passivated conventional semiconductors (e.g., Si or III–V compounds) (Scheuermann et al., 2013) or on low-cost metal oxides, such as hematite (Peter et al., 2012) or bismuth vanadate (Kim and Choi, 2014). An alternative, yet much less developed, concept is represented by “hybrid photoanodes” that comprise a “soft” molecular or polymeric light absorber supported on a wide-gap metal oxide acting as an electron collector and modified with an additional cocatalyst to promote the OER from water (Youngblood et al., 2009; Kirner et al., 2014; Ashford et al., 2015; Swierk et al., 2015; Kirner and Finke, 2017a, 2017b; Xu P. et al., 2017; Collomb et al., 2019; Zhang et al., 2019). A key advantage of this concept is that the wide-bandgap metal oxide support (e.g., TiO_2_) has typically a very negative potential of the conduction band edge, which alleviates the need for large external electric bias and makes the coupling with typical photocathodes in a tandem cell more feasible.

In this vein, we have investigated hybrid photoanodes based on nanocrystalline TiO_2_ electron collector modified with polymeric carbon nitride (“CN_x_,” also called polyheptazine or, more precisely, poly(aminoimino)heptazine or melon, a polymeric s-heptazine derivative, also referred to as “graphitic carbon nitride” or “g-C_3_N_4_” in the literature) (Wang et al., 2010), coupled with IrO_x_, CoO(OH)_x_ or NiO_x_ cocatalysts (Bledowski et al., 2012, 2013, 2014; Wang et al., 2012, Wang et al., 2017 L.; Mei et al., 2013; Khavryuchenko et al., 2015; Longchin et al., 2020). Such hybrid electrodes can be readily prepared by depositing CN_x_ into a mesoporous TiO_2_ anatase film on fluorine-doped tin oxide (FTO) glass by chemical vapor deposition from urea pyrolysis products. Since CN_x_-based materials typically suffer from low conductivity, the key advantage of such architectures is that the photogenerated electrons are collected by the TiO_2_ scaffold that provides a conductive transport pathway to the underlying FTO substrate. Interestingly, the resulting hybrid’s optical absorption edge was found to be extended into the visible range compared to pristine TiO_2_ (3.2 eV) or CN_x_ (2.9 eV), which we assigned to a direct optical electron transfer from the highest occupied molecular orbital (HOMO) of CN_x_ to the conduction band edge of anatase TiO_2_ (Bledowski et al., 2011). Importantly, we found out that the TiO_2_-CN_x_ hybrid required the presence of additional OER cocatalyst in order to induce complete water oxidation to dioxygen.

In the last decade (Liu and Aydil, 2009; Bang and Kamat, 2010; Chen et al., 2017; Shi et al., 2019; Yoon et al., 2019), single crystal TiO_2_ rutile nanorods (RNRs) electrodes have attracted a lot of interests because of the direct charge transport pathway and better electron mobility (Bang and Kamat, 2010) than that of conventional mesoporous electrodes fabricated from powders. These features make them also attractive for use as electron collectors in hybrid photoanodes. As an example, Wang et al. deposited CN_x_ on TiO_2_ RNRs and found an improved visible light photoactivity from CN_x_-RNR than both pure RNR and pure CN_x_ (Wang and Zhang, 2012). However, the surface area of the conventional RNR is limited due to the smooth surfaces (Wang et al., 2013). When used as the substrate for the immobilization of other photoactive materials (e.g., CN_x_), the small surface area is disadvantageous. Thus, many investigations have been done to modify the surface of RNRs and enlarge the surface area (Cho et al., 2011; Ye et al., 2013; Guo et al., 2014; Zhang et al., 2017).

In this study, we report a seed layer-free hydrothermal method, which enables the modification of the pure single crystalline RNR by polycrystalline anatase nanoparticles growing directly on the RNR surfaces. The nanocrystalline anatase layer on the rutile nanorods is found to be of crucial importance as after deposition of CN_x_, the resulting anatase-wrapped RNR (ARNR) electrodes show enhanced visible light absorption as compared to the RNR electrodes. Moreover, after deposition of CoO(OH)_x_ as OER cocatalyst (Wang L. et al., 2017), the photoelectrocatalytic activity of CN_x_-ARNR is improved as well, generating more oxygen than its CN_x_-RNR counterpart under visible light irradiation in a PEC cell.

## Materials and Methods

### Materials

Tetrabutyl titanate, TiCl_3_ (20% dissolved in 2 N HCl), was purchased from Acros Organics. 2-Propanol, acetone, urea, 37% HCl, cobalt (II) nitrate hexahydrate, 25% ammonia solution, sodium hydroxide, and boric acid were provided by Sigma-Aldrich. Fluorine-doped tin oxide (FTO) Pilkington TEC glass (3 mm × 15 mm × 25 mm) was purchased from XOP company (Xop Glass, Castellón Spain). FTO glass was cleaned with 2-propanol, deionized water, and acetone consecutively in an ultrasonic bath before usage. Deionized water was used for the synthesis and rinsing of samples.

### Synthesis

#### Rutile Nanorod Electrode Preparation

RNR arrays were prepared according to an adapted method from the literature (Cho et al., 2011), but without using a seed layer. Briefly, 9 ml of 37% HCl was mixed with 9 ml H_2_O and stirred for 5 min. Then, 0.2 ml tetrabutyl titanate was added and stirred till a transparent solution was obtained. The solution was transferred into a Teflon-lined autoclave with a piece of FTO glass placed at an angle around 60° inside. The autoclave was heated to 170°C with a rate of 5°C/min and kept at 170°C for 6 h. After the autoclave was cooled down to room temperature in air, the as-prepared RNR was taken out, deeply rinsed with deionized water, and dried in air. RNR was calcined at 450°C for 30 min in air before CN_x_ deposition.

#### Anatase-Wrapped Rutile Nanorod Electrode Preparation

The rinsed as-prepared RNR was directly used for ARNR preparation also without any seed layers. Briefly, 0.02 ml of 37% HCl and 0.2 ml TiCl_3_ (dissolved in 2 N HCl) were mixed with 17 ml H_2_O and quickly transferred into a Teflon-lined autoclave with the RNR placed at an angle around 60° inside. The autoclave was heated to 80°C and kept at 80°C for 2 h. After the autoclave was cooled down to room temperature in air, the as-prepared ARNR was taken out, deeply rinsed with deionized water, and dried in air. Finally, the ARNR was calcined at 450°C for 30 min in air.

#### CN_x_ Deposition

The as-prepared electrodes were modified by polymeric carbon nitride by vapor deposition from urea according to the method developed by our research group (Wang L. et al., 2017). First, two electrodes of RNR or ARNR were placed into a Schlenk tube connected via an adapter with a round-bottom flask containing 1 g of urea. Afterward, the loaded reactor was transferred into a preheated muffle oven (425°C). After 30 min at 425°C, the reactor was removed from the oven and cooled in air.

#### CoO(OH)_x_ Cocatalyst Deposition

CoO(OH)_x_ was deposited by the impregnation method reported by us previously (Wang L. et al., 2017). The CN_x_-containing electrode was firstly immersed in 0.1 M Co(NO_3_)_2_ aqueous solution for 10 min. After being dried in air, the electrode was quickly dipped into 25% ammonia solution and dried in air again.

### Photoelectrocatalytic Measurements

The photoelectrochemical measurements were conducted using SP-300 BioLogic potentiostat and a typical three-electrode system consisting of a Pt wire counter electrode, an Ag/AgCl (3.5 M KCl, 0.207 V vs. SHE) reference electrode, and the RNR or ARNR-based photoanode working electrodes with geometric irradiation area of 0.5 cm^2^. A 150 W Xe lamp (L.O.T.-Oriel) with a light power density of ∼180 mW/cm^2^ equipped with a KG-3 (LOT-Quantum Design) heat-absorbing filter and 420 nm longpass optical filter was used. All electrodes were illuminated from the backside (through FTO glass) as the photocurrents were higher than when irradiated from the front side. The oxygen evolution was recorded by FireSting optical fiber oxygen meter (PyroScience, GmbH) in a homemade air-tight two-compartment cell. The oxygen concentrations are measured in the solution and are not corrected for the losses in the gaseous headspace; the oxygen collection efficiency of approximately 75% was estimated by direct electrolysis using a Pt working electrode. The volume of the photoanode compartment was 5 ml. The electrolyte was purged by Ar before the electrodes were illuminated under an applied potential of 1.12 V versus RHE. The incident monochromatic photon-to-current conversion efficiency (IPCE) was recorded using a photoelectric spectrometer (Instytut Fotonowy Sp. z o.o.) equipped with a tunable monochromatic light source provided with a 150 W Xenon lamp and a grating monochromator with a bandwidth of ∼10 nm. Photoaction spectra were measured at 1.12 V versus RHE in a borate solution (0.1 M, pH 8). The value of photocurrent density was the difference between current density under irradiation and in the dark in steady-state conditions with a wavelength sampling interval of 10 nm. The IPCE value for each wavelength was calculated according to the following equation:

$$\mathrm{IPCE}\left(\%\right)=\left(i_{\mathrm{ph}}\mathrm{hc}\right)/\left(\mathrm{\lambda Pq}\right)\times 100\%,$$

where *i*_ph_ is the photocurrent density, *h* is Planck’s constant, *c* is the velocity of light, *P* is the light power density, *λ* is the irradiation wavelength, and *q* is the elementary charge. The electrolyte for all photoelectrochemical measurements was 0.1 M sodium borate solution with a pH value of 8, unless stated otherwise. All potentials are recalculated and reported versus Reversible Hydrogen Electrode (RHE).

### Characterization

The optical properties were investigated by the UV-Vis spectrophotometer (UV-2600, Shimadzu, Japan) equipped with the integrating sphere and the absorptance (Abs.) was calculated by the equation Absorptance (%) = 100% − Reflectance (%) − Transmittance (%). Powder X-ray diffraction (XRD) patterns were recorded on the diffractometer (XRD-6000, Rigaku, Japan) under the following conditions: 40 kV, 40 mA, CuKα radiation (*λ* = 0.154 nm). Photoluminescence (PL) was recorded using an RF-6000 (Shimadzu, Japan) spectrofluorophotometer with the excitation wavelength of 360 nm; a 400 nm cut-off filter was placed in front of the emission monochromator. Morphological analysis was performed by a FIB/SEM (focused ion beam, scanning electron microscope) instrument (Carl Zeiss NVision 40). Internal morphology was acquired by milling the samples with the Ga-ion beam (FIB) in the instrument chamber. The elemental composition was investigated by energy-dispersive X-ray (EDX) spectroscopy (EDAX Octane Elite) of the FIB/SEM. For TEM imaging, a cross-sectional sample of the ARNR was prepared by mechanical grinding and dimpling, followed by Ar-ion milling to obtain an electron transparent area. High-resolution TEM imaging was then performed using an image-side aberration corrected FEI Titan 80–300 microscope operated at an acceleration voltage of 300 kV. Scanning transmission electron microscopy (STEM) imaging together with local EDX spectroscopy was carried out using a Thermo Fisher Talos 200X equipped with a SuperX X-ray detector at 200 kV operation voltage.

## Results and Discussions

**FIGURE 1:**
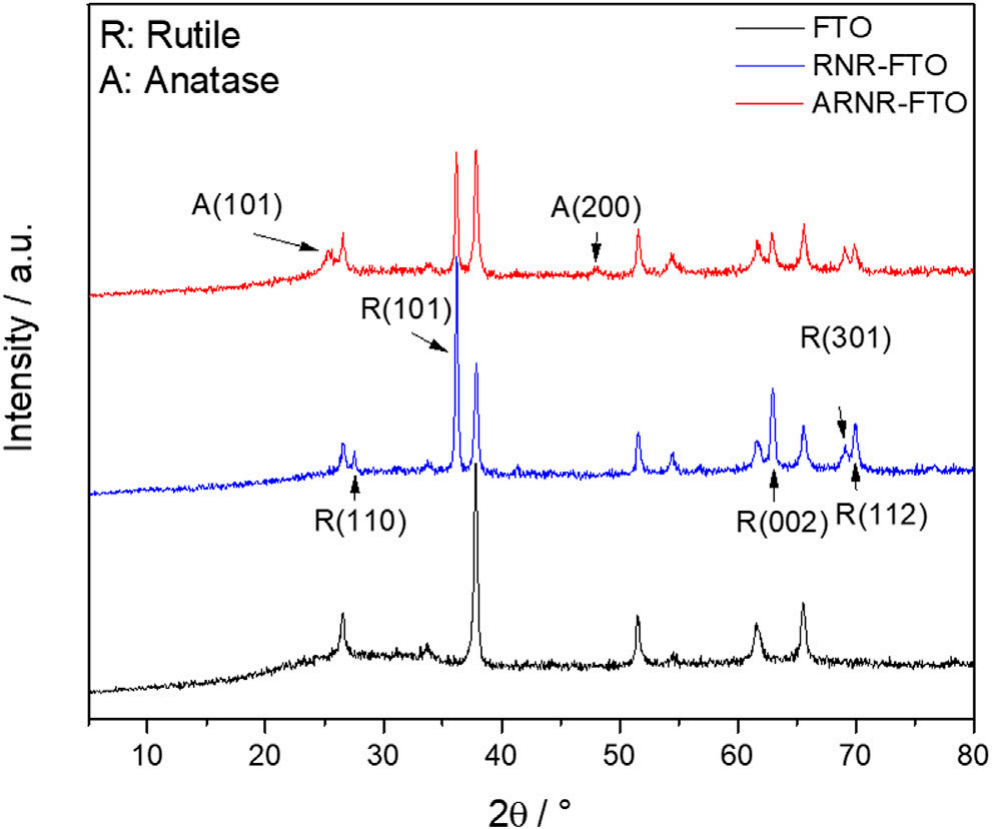
XRD patterns of RNR, ARNR, and of the FTO substrate. Reflections assigned to anatase and rutile are abbreviated with A and R, respectively.

It is well established in the literature that hydrothermal treatment of rutile nanorods with TiCl_3_ typically leads to branched rutile TiO_2_ nanorods comprised of nanorod trunks and nano-branches, both of pure rutile phases (RNR) (Cho et al., 2011; Xu F. et al., 2017; Wang Y. et al., 2017). Herein, we show, for the first time, that an adjusted TiCl_3_ treatment results in a distinct type of decorated nanorod arrays (ARNRs) in which rutile nanorods are wrapped by a thin anatase nanoparticulate layer. It is also worth noting that both RNR and ARNR in this study are synthesized by hydrothermal reactions without any seed layers (details in the section of materials and methods). The XRD pattern of the conventional RNR shows characteristic XRD reflection peaks of rutile only (Figure 1). The most intensive peak at 2θ ∼38.1° matches the (101) crystal plane, which indicates the main growing orientation of RNR as [010]. After being treated with TiCl_3_, two new XRD peaks of anatase at 25.5° and 48° were detected, while the low-intensity peak from (110) of rutile became non-detectable, which was probably caused by the evolution of anatase.

**FIGURE 2:**
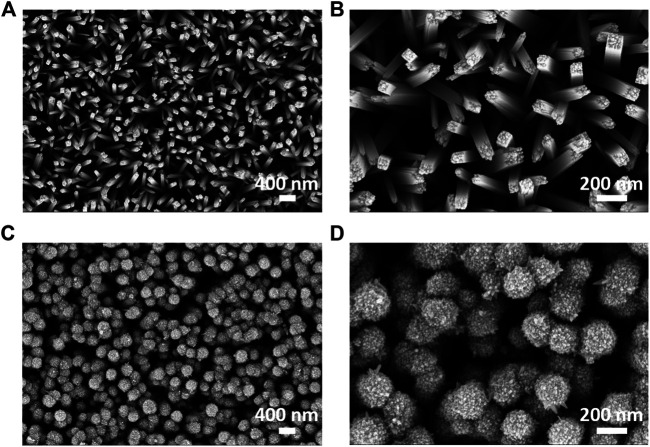
Top-view SEM images: **(A,B)** RNR and **(C,D)** ARNR.

In order to investigate the morphology of RNR and ARNR materials, scanning electron microscopy was employed. Figures 2A,B are the top-view SEM images of RNR at lower and higher magnifications. The morphology of the RNR in our study is similar to the RNR from the literature (Liu and Aydil, 2009) with a tetrahedral prism shape and smooth surfaces. The average width and length of RNR here are estimated as ∼100 nm and ∼2 μm, respectively. In contrast, the hydrothermal treatment of RNRs with TiCl_3_ leads clearly to ARNR nanorods with a larger diameter and rougher morphology (Figures 2C,D). The cross-section SEM image of ARNR material (Supplementary Figure S1) reveals a gradual morphological change from the bottom (interface with FTO) to the top of the nanorod. The TiCl_3_ treatment is clearly responsible for the rough structure predominantly on the top of RNR, whereas the smooth and tetrahedral prism morphology is retained close to the substrate. Furthermore, the elemental composition of the sample at a cross-section was confirmed by means of scanning transmission electron microscopy in combination with energy-dispersive X-ray spectroscopy (STEM-EDX) (Supplementary Figure S2).

**FIGURE 3:**
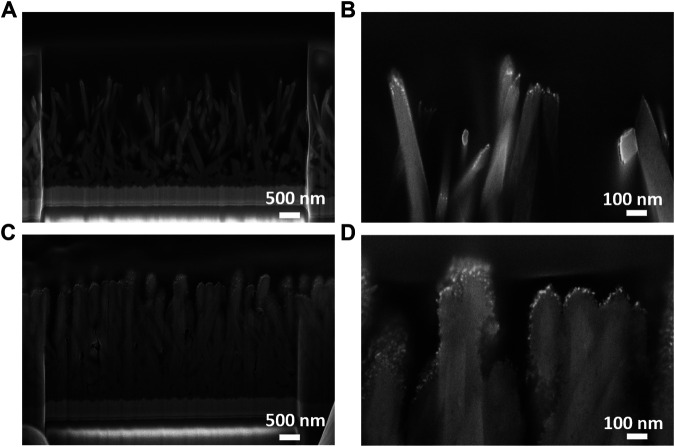
Cross section SEM images: **(A,B)** RNR and **(C,D)** ARNR milled by FIB.

With the aim of examining the internal structure of the bare and anatase-modified rutile nanorods, the cross sections of both materials were also studied in detail using focused ion beam (FIB) milling combined with SEM (Figure 3). The compact internal structure was found for both types of nanorods, while in case of ARNR, it is evident that the anatase phase is wrapping around the upper part of the rutile nanorod trunk with a smooth boundary between the phases. Furthermore, a higher roughness and larger surface area of the ARNR, compared to RNRs, are confirmed.

**FIGURE 4:**
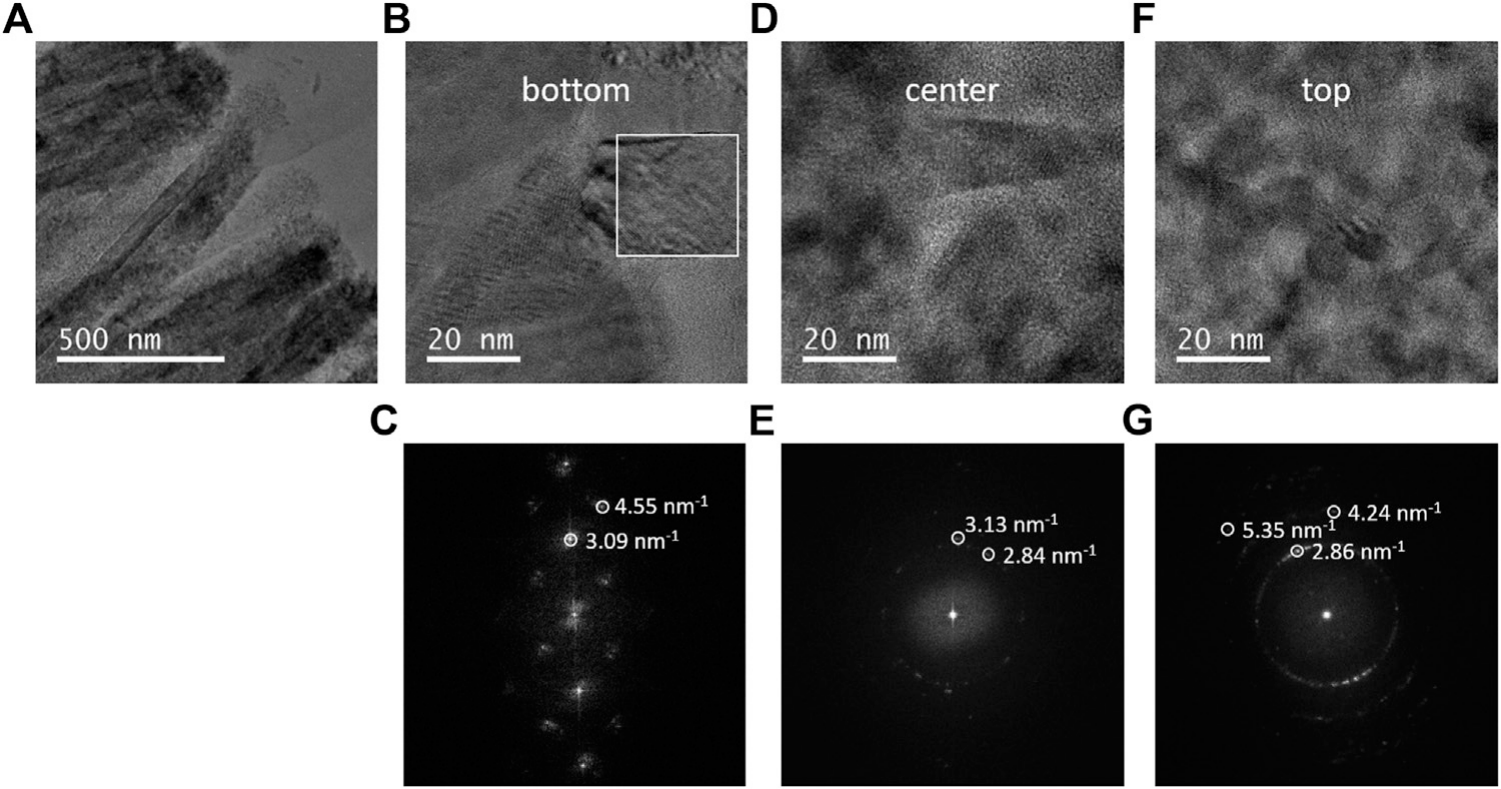
**(A)** HRTEM images of ARNR at lower magnification. **(B,D,F)** HRTEM images of different locations of ARNR at higher magnification. **(C,E,G)** Fast Fourier transform (FFT) of different locations of ARNR.

In order to gain in-depth information on the morphology and precise phase type and phase distribution of the ARNR, we studied the lower, middle, and upper part of the nanorod by high-resolution transmission electron microscope (HRTEM) and electron diffraction (Figure 4). The HRTEM image of the ARNR cross section at a medium magnification supports our former findings that the rutile nanorod trunks are surrounded by a nanoparticle shell with thickness gradually increasing from the middle to the top of the nanorod (Figure 4A). Finally, HRTEM images along the mixed phase nanorod were recorded at high magnification (Figures 4B,D,F) and corresponding diffractograms obtained by applying a 2D Fourier transform are depicted in Figure 4C,E,G. The diffractogram of the shell-free part of the nanorod trunk is characteristic of single crystalline rutile (Figure 4C). Near the center of the nanorod, the diffractogram shows numerous spots from anatase at different orientations and spots originated from rutile, which indicate the presence of polycrystalline anatase in addition to the rutile trunk (Figure 4E). The Fourier transform from the top part of the ARNR also confirms the presence of anatase in many different orientations (Figure 4G), while the rutile phase was not detected. The nanocrystalline anatase domains observed here are in line with the XRD pattern of the anatase phase in ARNR (Figure 1) which is s characteristic of low-intensity and broad peaks due to nanoscopic dimensions of these anatase crystallites. All the analyses above confirm the distinct morphology of ARNR as anatase-wrapped rutile nanorods.

**FIGURE 5:**
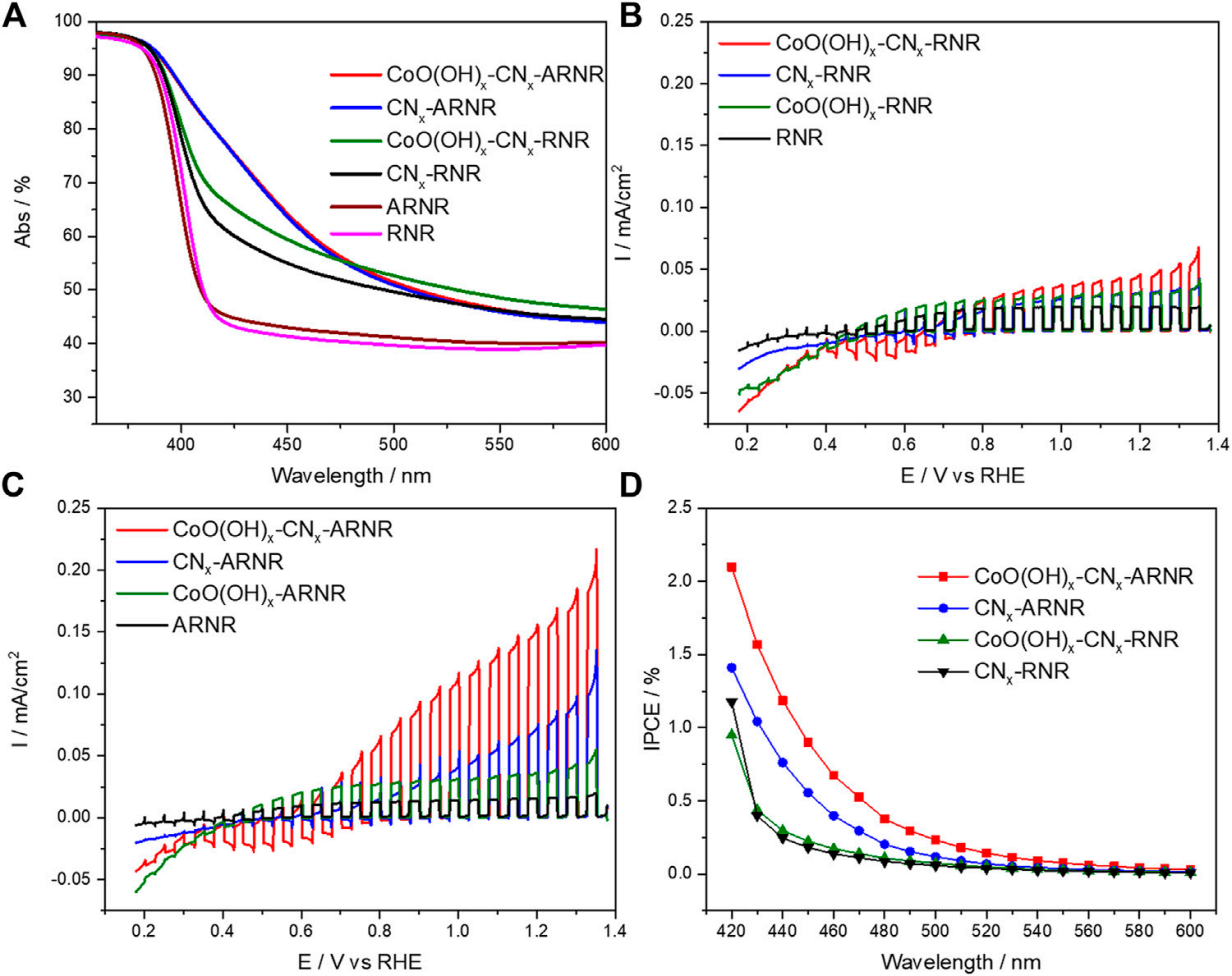
Optical properties and photoelectrocatalytic characterization: **(A)** UV-Vis absorptance spectra. **(B)** Photocurrent transients recorder in borate solution (0.1 M, pH 8) under visible light irradiation *λ* > 420 nm during cathodic potential sweep (5 mV/s) at RNR-based electrodes and **(C)** ARNR-based electrodes. **(D)** Photoaction spectra (IPCE) recorded under intermittent monochromatic irradiation at 1.12 V versus RHE (0.1 M sodium borate, pH 8). The nonzero baseline of absorptance spectra **(A)** is attributed to the internal reflection and scattering at the FTO/TiO_2_ interface leading to photons exiting from the measuring pathway and avoiding detection (Cho et al., 2011). In the presence of the CoO(OH)_x_ cocatalyst, the positive photocurrents near the onset potential (in B and C) are overlaid by dark negative currents which are most likely related to the electrochemical reduction of the cobalt cocatalyst that is in contact with FTO.

In order to evaluate the performance of the titania nanorod structures as electron collectors in a hybrid photoanode for oxygen evolution from water under visible light irradiation, we have modified bare and anatase-wrapped nanorods with the polymeric carbon nitride photoabsorber, followed by CoO(OH)_x_ cocatalyst deposition. The absorptance spectra of unmodified RNR and ARNR samples are very similar, as corroborated by the bandgap energies of 3.0 eV for both samples (Figure 5A, Supplementary Figure S9), a bandgap value typically reported for pure rutile (Syarif et al., 2002). On the other hand, after modification with CN_x_, the CN_x_-ARNR electrode showed a redshift of the absorption edge (∼2.5 eV) as compared to the anatase-free CN_x_-RNR sample (2.9 eV) (Figure 5A, Supplementary Figures S3, S9). This result indicates that the enhanced visible light absorption is due to the presence of anatase nanoparticles which provide a larger surface area for effective CN_x_ deposition than bare rutile nanorod. This is not surprising as it is known that the formation of CN_x_ from urea pyrolysis products is catalyzed by OH groups at the TiO_2_ surface (Mitoraj and Kisch, 2008). The presence of nitrogen and carbon in both materials is confirmed by elemental EDX spectra (Supplementary Figure S4).

It is also noteworthy that the CN_x_ deposition on ARNR or RNR did not change the XRD patterns (Supplementary Figure S5) and the morphologies observed in the SEM images (Supplementary Figure S6) of both electrodes. Finally, after deposition of the cocatalyst, the absorption spectrum of CoO(OH)_x_-CN_x_-ARNR remains the same as the spectrum of CN_x_-ARNR suggesting the absence of the undesired parasitic light absorption by the CoO(OH)_x_ cocatalyst (Figure 5A). The presence of CoO(OH)_x_ on CN_x_-ARNR and CN_x_-RNR is further evidenced by EDX elemental spectra in addition to CN_x_ (Supplementary Figure S7). Meanwhile, no bulky Co-containing species is detected from EDX mappings (Supplementary Figure S8), which confirms a homogeneous distribution of ultra-small (∼1–2 nm) CoO(OH)_x_ nanoparticles, in line with our previous study (Wang L. et al., 2017).

As a next step, the photocurrent response of the photoanodes was evaluated by potential-dependent photocurrent transient measurements in 0.1 M borate electrolyte of pH 8 under chopped visible light illumination (*λ* > 420 nm). As depicted in Figures 5B,C, the poor photocurrent densities recorded at bare rutile and anatase/rutile nanorods are comparable and originate from the intrinsic photoresponse of TiO_2_. As expected, the photocurrents are slightly improved after CoO(OH)_x_ particles deposition. On the other hand, enhanced photocurrent density for the cocatalyst-free nanorods containing a polymeric absorber is attributed to the oxidation of the CN_x_ layer. Notably, higher photocurrent for CN_x_-anatase/rutile nanorods arises from improved visible light absorption as compared to the CN_x_-rutile nanorods (Figures 5B,C, blue curve). Finally, photocurrent increases after coupling of the nanorods with the CoO(OH)_x_ particles, which is attributed to the enhanced hole extraction from the organic layer to the cocatalyst and to triggering of water oxidation to dioxygen, as discussed below. The chronoamperometric (CA) study focusing on the ARNR-based electrodes (Supplementary Figure S10) also provides evidence for the improved hole extraction from CoO(OH)_x_-CN_x_-ARNR compared with CN_x_-ARNR that exhibits a sharp spike when the light is switched on and an overshoot current after the light is switched off. Both phenomena reveal the severe charge recombination and hole accumulation in CN_x_ when CoO(OH)_x_ is absent (Bledowski et al., 2014; Ye et al., 2018). Evidently, a higher concentration of holes photogenerated in ARNRs due to the higher amount of CN_x_ absorber translates into higher photocurrent density than for the anatase-free nanorods. On the other hand, the photogenerated electrons are collected at the titania as supported by photocurrent onset potential for the photoanodes of ∼0.2 V being close to the conduction band edge of titania (–0.15 V vs. RHE (Kavan et al., 1996)). In addition, the effects of electrolyte and pH value are also studied under otherwise identical experimental conditions. As expected, lower photoactivity of CoO(OH)_x_-CN_x_-ARNR photoelectrode especially at lower potentials was obtained in sodium sulfate electrolyte with the same pH of 8 as in borate (see Supplementary Figure S11). We assume that sulfate ions, as harder bases than borate ions in this pH range, interact more strongly with Co(III/IV)-ions (i.e., hard acids) produced during photoelectrocatalysis in CoO(OH)_x_, rendering thus the latter less stable (Wang L. et al., 2017). Notably, the photoelectrocatalytic performance of CoO(OH)_x_-CN_x_-ARNR (see Supplementary Figure S12) is significantly lower at a lower pH of 7 in sodium borate electrolyte. This strong pH sensitivity is probably attributed to the slower water oxidation kinetics of CoO(OH)_x_ in neutral electrolytes (Wang L. et al., 2017), indicating that the interfacial charge transfer to water molecules represents the performance-determining step for this water-splitting system.

**FIGURE 6:**
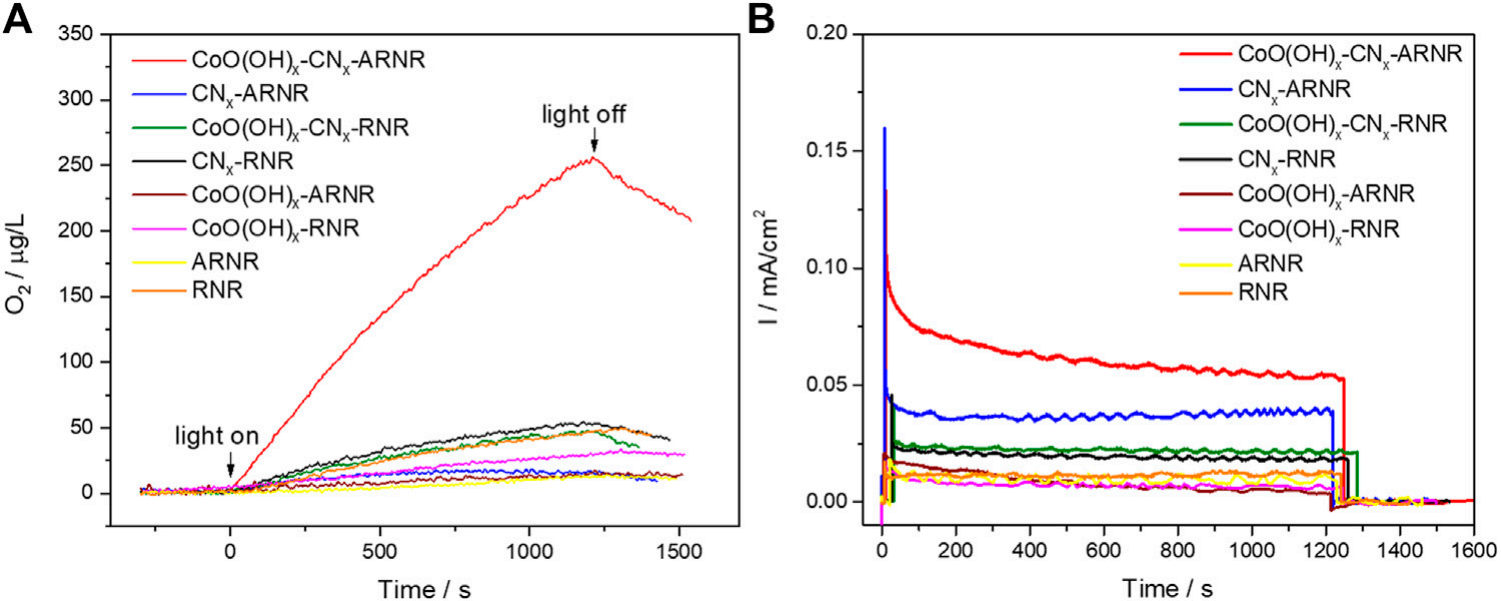
**(A)** OER curves of ARNR and RNR-based electrodes under visible light irradiation (λ > 420 nm) measured at 1.12 V versus RHE (0.1 M borate, pH 8). **(B)** Corresponding photocurrent transients.

Incident photon-to-current conversion efficiency (IPCE, Figure 5D and the simultaneous transient photocurrent, Supplementary Figure S13) was measured upon monochromatic irradiation from the substrate side in 0.1 M borate solution at pH 8. The incident quantum efficiencies are clearly improved upon cocatalyst deposition at CN_x_-anatase/rutile nanorods. This suggests a more efficient extraction of holes which can be now utilized for water oxidation to dioxygen (see Figure 6), as corroborated by the highest photocurrents upon polychromatic visible light irradiation of this photoanode (Figure 5C). Notably, the long shoulder of the IPCE values into the red of the photoaction spectrum lines up with the absorption spectrum for the CoO(OH)_x_-CN_x_-ARNRs, as also indicated by the absorbed photon-to-current conversion efficiency photoaction spectrum (APCE, Supplementary Figure S14). In the absence of the cocatalyst, the measured efficiencies are likely dominated by the oxidative photocorrosion of the CN_x_. For rutile based RNR photoanodes, only negligible IPCE values were observed primarily due to poor absorption of the visible light.

The photoluminescence (PL) spectra were measured for all CN_x_-containing electrodes (Supplementary Figure S15) to compare the charge recombination between catalyst-containing electrodes and catalyst-free electrodes. The PL measurements were also performed at bare RNR, bare ARNR, and FTO. Comparable PL intensities for CN_x_-RNR and CoO(OH)_x_-CN_x_-RNR were obtained suggesting similar dynamics of the charge recombination and separation in both materials. This is in accordance with the similar photocurrent and IPCE values of these two RNR-based electrodes. On the other hand, PL intensity significantly drops at ca. 475 nm and does not change at ca. 575 nm from CN_x_-ARNR and CoO(OH)_x_-CN_x_-ARNR, respectively. We attribute the PL band with a maximum at about 475 nm to the luminescence of the bulk carbon nitride whereas the PL band at about 575 nm to the emission due to CN_x_/anatase interface, since the latter peak is absent in CN_x_-RNR and bare ARNR. We conclude that CoO(OH)_x_ cocatalyst is responsible for improved charge collection from the carbon nitride layer, while recombination at CN_x_/anatase interface is not influenced. With this respect, the suppressed recombination rate likely contributes to the higher photocurrent and IPCE values for CoO(OH)_x_-CN_x_-ARNR as compared to other photoelectrodes.

**FIGURE 7:**
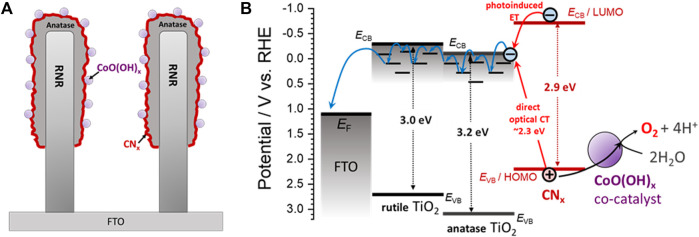
A simplified representation **(A)** and energy band diagram **(B)** for a CoO(OH)_x_-CN_x_-ARNR photoanode investigated in this work. The offset of the conduction band edges at the rutile/anatase interface is taken as 0.3 eV based on the literature data (typically ca. 0.1–0.5 eV) (Deák et al., 2011; Pfeifer et al., 2013; Scanlon et al., 2013; Kullgren et al., 2015; Mansfeldova et al., 2021). Since we did not observe any intrinsic effect of the rutile/anatase interface on the charge separation (see Supplementary Figure S16), we assume that the charge separation and transport are not significantly influenced by the offset of the conduction band edges of rutile and anatase, which might be due to the strong influence of the environment (CN_x_ and aqueous electrolyte) on the interfacial energetics (Kullgren et al., 2015; Mansfeldova et al., 2021) and/or the effect of trap states under the conduction band edge of TiO_2_ (Berger et al., 2012). A drift-diffusion transport of photogenerated electrons through anatase and rutile is assumed to occur *via* thermally activated trap-assisted hopping (Nelson, 1999), as indicated by the blue arrows. *E*_CB_ and *E*_VB_ stand for the conduction and valence band edges; *E*_F_ stands for the Fermi level of the FTO.

Finally, in order to estimate the visible light-driven OER performance of the studied electrodes, dioxygen evolution in 0.1 M borate electrolyte was recorded during electrode irradiation with the visible light (*λ* > 420 nm; see Figure 6). On one hand, each RNR-based electrode only produced a negligible amount of O_2_ presumably due to the limited visible light absorption. On the other hand, among the ARNR-based electrodes, only CoO(OH)_x_-CN_x_-ARNR photoanode showed superior OER performance in contrast to the ARNR, CN_x_-ARNR, and CoO(OH)_x_-ARNR. Here, CN_x_-ARNR did not produce any O_2_ despite its stable photocurrent, which is due to the photocorrosion of CN_x_ in the absence of cocatalyst. In addition, the CoO(OH)_x_-CN_x_-ARNR showed a faradaic efficiency of ∼41.5%, which doubles that of CoO(OH)_x_-CN_x_-RNR (∼20.6%). Clearly, not all photogenerated holes oxidize water to dioxygen but a significant portion of them induces other oxidative processes, most probably the oxidative photodegradation of CN_x_. However, our results indicate that the CoO(OH)_x_-CN_x_-ARNR photoanode not only separates the charges more efficiently but also utilizes a higher number of the photoholes from CN_x_ for the OER as compared to the CoO(OH)_x_-CN_x_-RNR. Hence, it can be concluded that the three components, ARNR (an effective electron collector and substrate for CN_x_ deposition), CN_x_ (visible light absorber), and CoO(OH)_x_ (cocatalyst), are all indispensable for the superior OER performance of the best photoelectrode (Figure 7).

## Conclusion

Arrays of single crystal TiO_2_ rutile nanorods (RNRs) appear highly promising as electron-collecting substrates in hybrid photoanodes as the RNRs offer direct charge carriers transport pathways, contrary to the conventional electrodes prepared from TiO_2_ powders that suffer from the numerous charge traps at the grain boundaries. However, the specific surface area of the nanorods is limited by their smooth morphology, which is detrimental in view of utilizing the RNR as a substrate for immobilizing other functional materials. In this work, we developed novel anatase-wrapped rutile nanorods (ARNRs) arrays using a facile seed layer-free hydrothermal method and demonstrated their superior performance as electron collectors in hybrid photoanodes for water oxidation by visible light. The nanocrystalline anatase layer on the rutile nanorods in ARNR is found to be of crucial importance for effective deposition of polymeric carbon nitride and thus for effective photoanode operation in visible light-driven (*λ* > 420 nm) oxygen evolution. This study highlights the importance of careful interfacial engineering in constructing photoelectrocatalytic systems for solar energy conversion and paves the way for the use of ARNR-based electron collectors in further hybrid and composite photochemical architectures for solar fuel production.

## Data Availability

The original contributions presented in the study are included in the article/Supplementary Material; further inquiries can be directed to the corresponding authors.
