# Supplementary material for: Anatase-Wrapped Rutile Nanorods as an Effective Electron Collector in Hybrid Photoanodes for Visible Light-Driven Oxygen Evolution
Source: Front Chem. 2021 Aug 18;9:709903. doi: 10.3389/fchem.2021.709903 (PMC8416449; doi:10.3389/fchem.2021.709903)
Supplement: Supplementary file 1 [file DataSheet1.pdf]

Supplementary Material

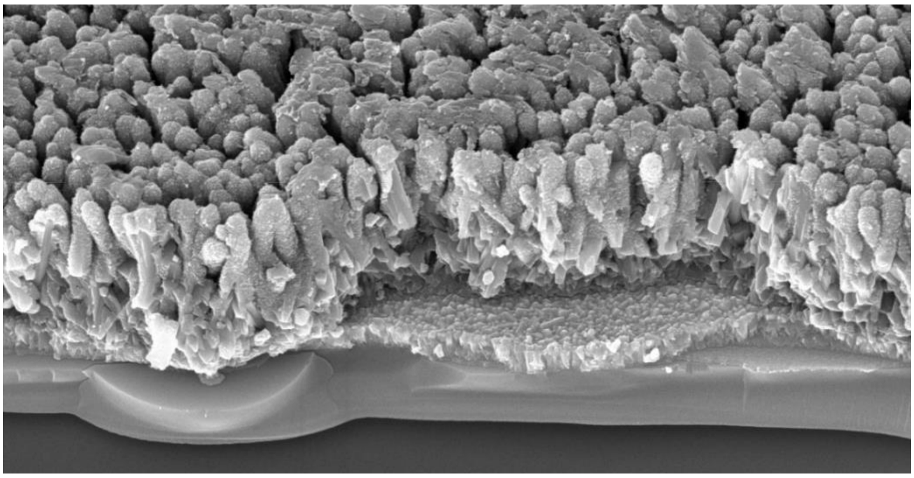

Figure S1 Cross-section SEM image of ARNR.

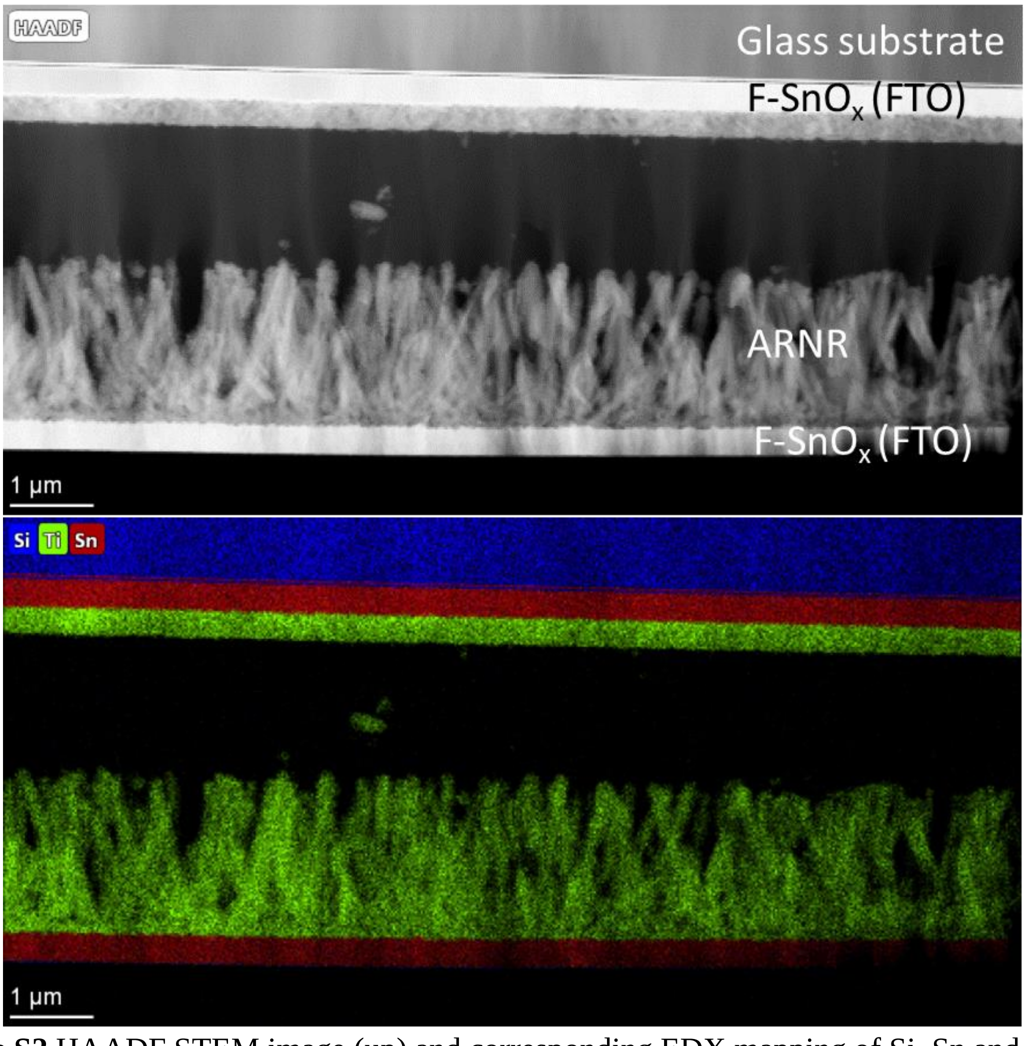

Figure S2 HAADF STEM image (up) and corresponding EDX mapping of Si, Sn and Ti (down), showing an overview of the cross-section of ARNR sample. The substrate is silicon oxide glass, covered with a 300 nm thick fluorine doped tin oxide layer (FTO). The ARNR are roughly 2 μm long.

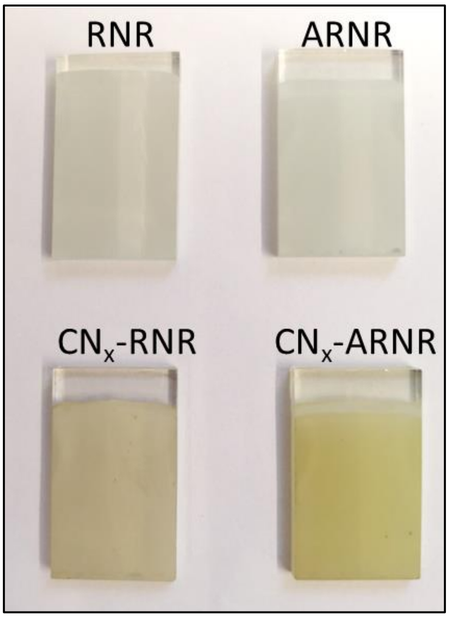

Figure S3 Optical photograph of RNR, ARNR, CN_x-RNR, CN_x-ARNR.

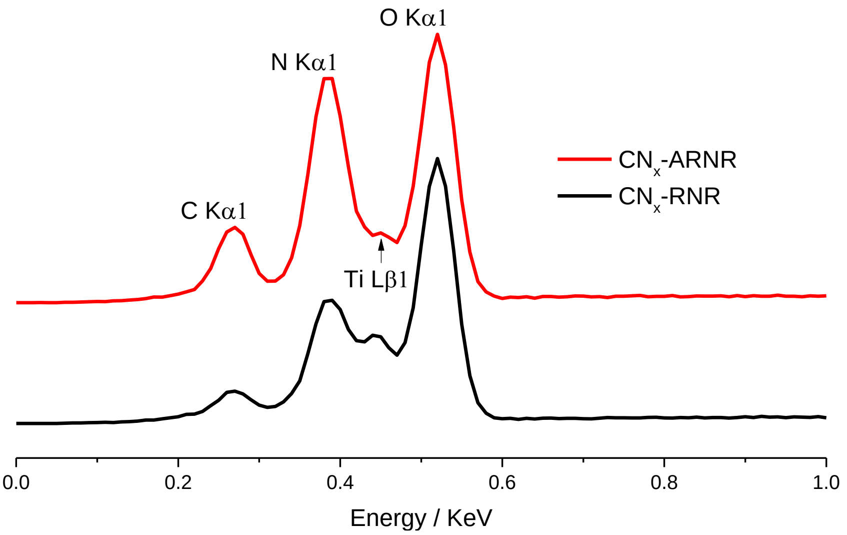

Figure S4 Elemental EDX spectra of CN_x-ARNR and CN_x-RNR.

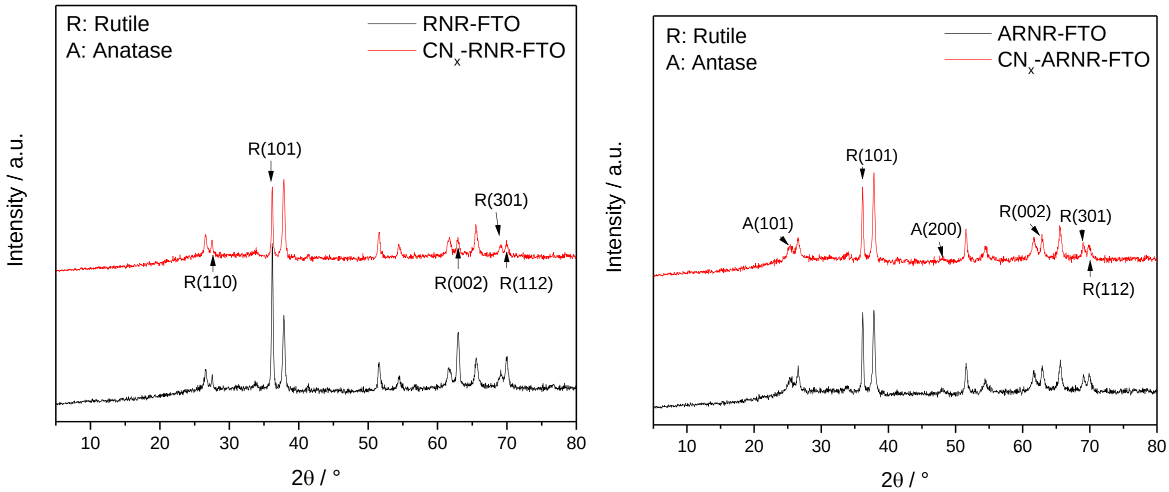

Figure S5 XRD patterns of RNR and CN_x-RNR (left), XRD patterns of ARNR and CN_x-ARNR. Peaks assigned to anatase and rutile are abbreviated with A and R, respectively.

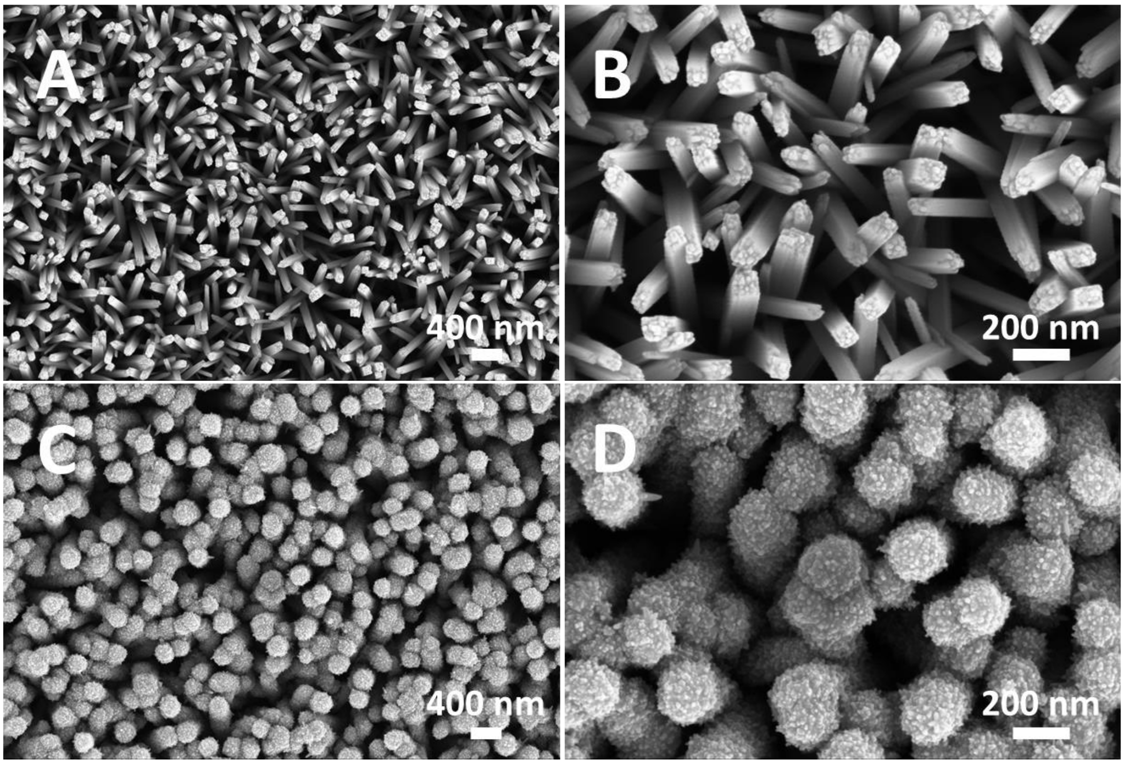

Figure S6 SEM images of CN_x-RNR **(A, B)** and CN_x-ARNR **(C, D)**.

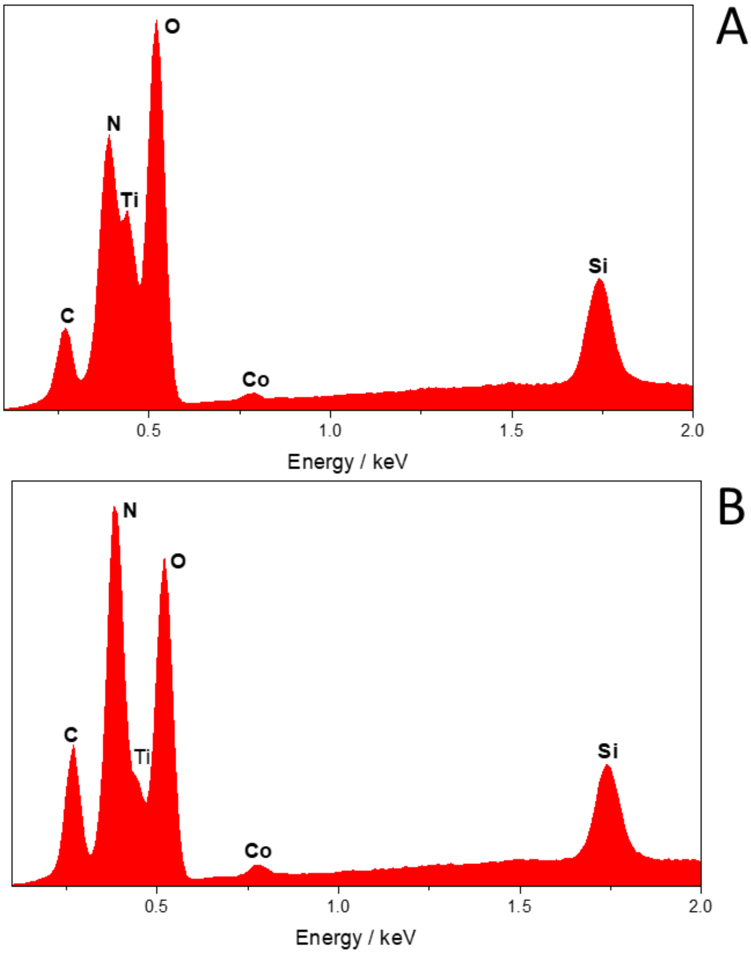

Figure S7 EDX elemental spectra of $CoO(OH)_x$-CN_x-RNR **(A)**, and $Co(OH)_x$-CN_x-ARNR **(B)**.

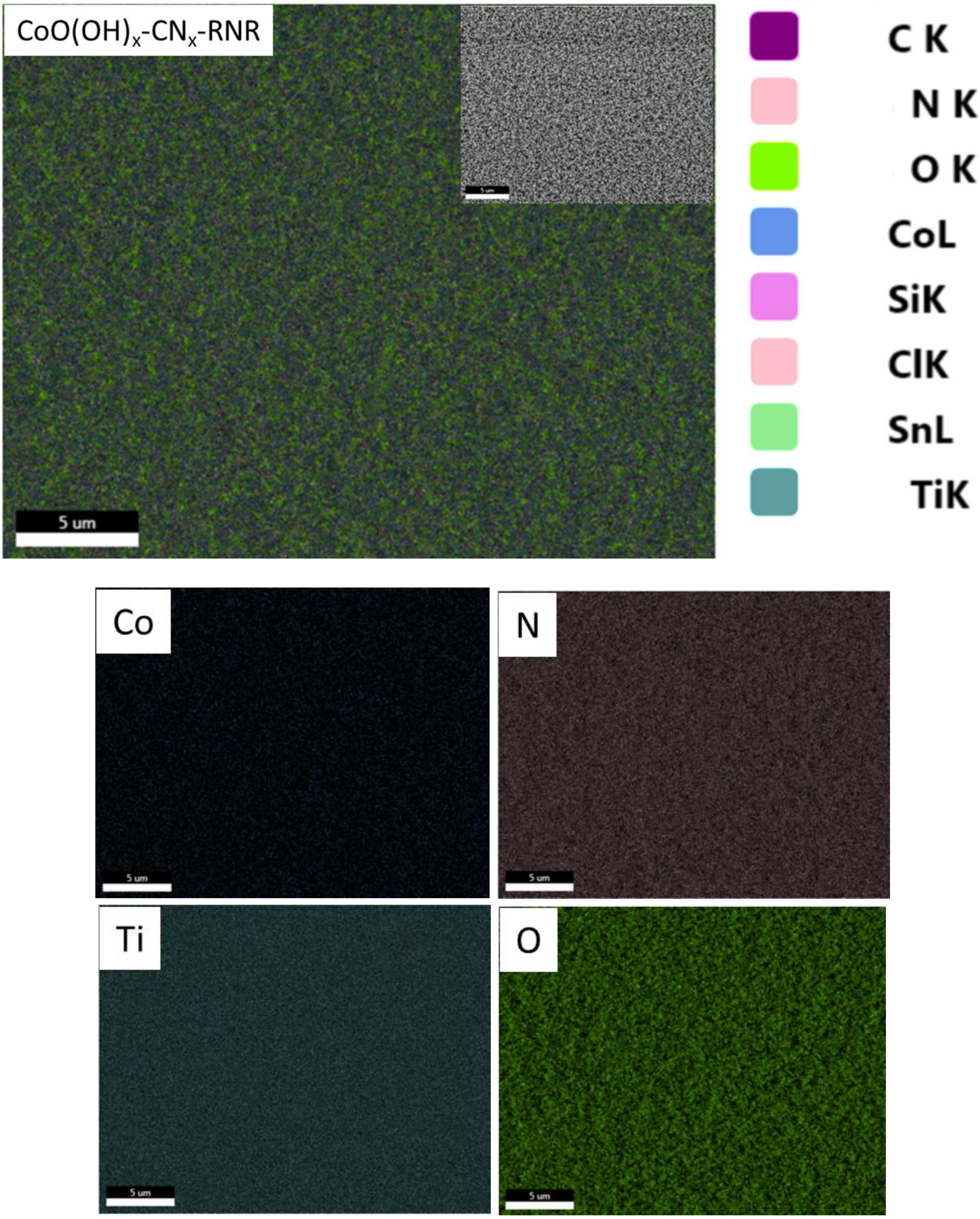

$CoO(OH)_x$-CN_x-RNR
5 um
C K
N K
O K
CoL
SiK
ClK
SnL
TiK
Co
N
Ti
O

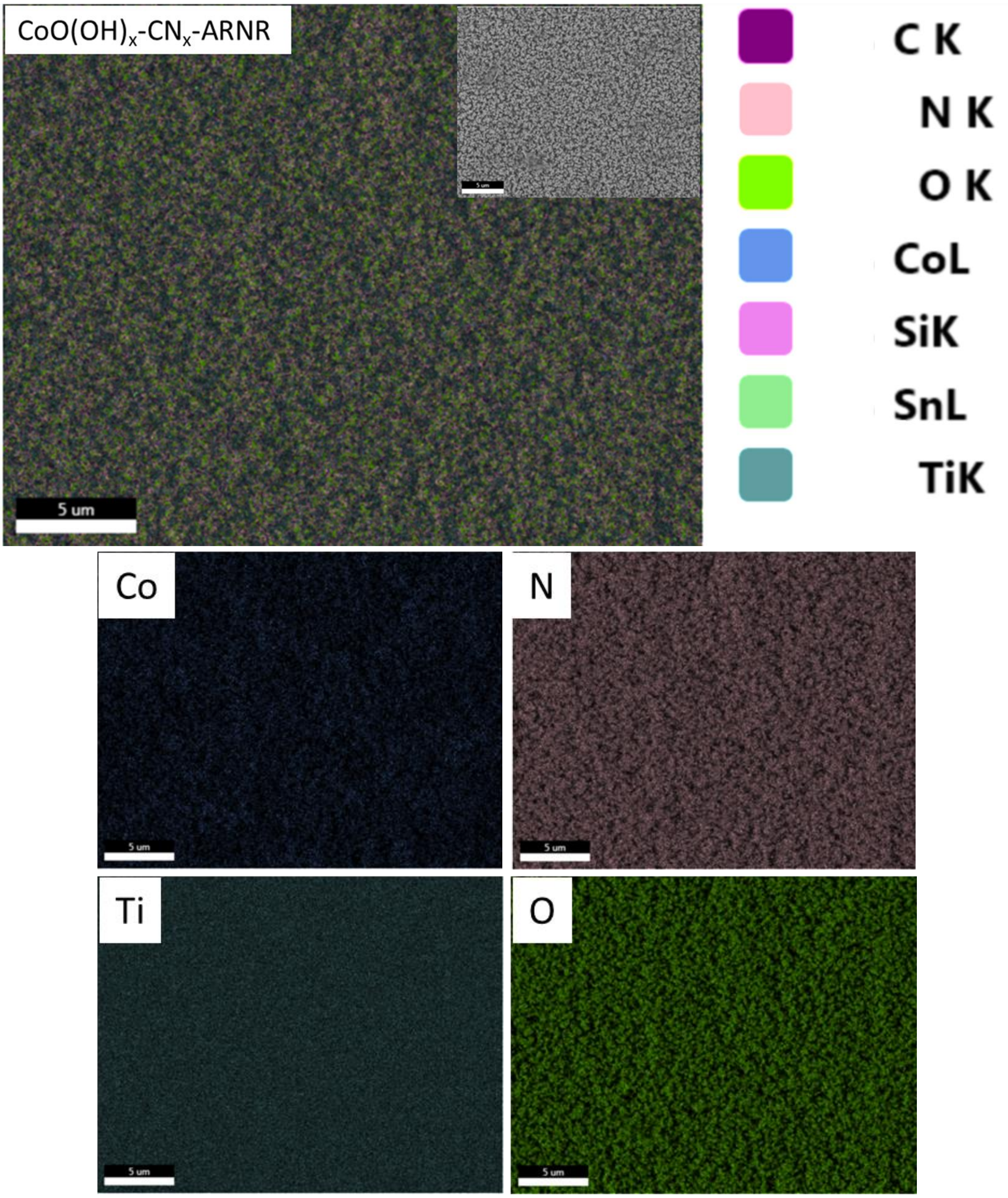

Figure S8 EDX mappings and corresponding SEM images of the investigated areas (inset) of $CoO(OH)_x$-CN_x-RNR and $CoO(OH)_x$-CN_x-ARNR. A homogeneous distribution of all elements is observed. The Co content estimated from three different spots was 1.7±2.2 wt% and 0.3±0.7 wt% for $CoO(OH)_x$-CN_x-ARNR and $CoO(OH)_x$-CN_x-RNR, respectively (errors taken as double of the standard deviation; *i.e.*, 95% confidence interval).

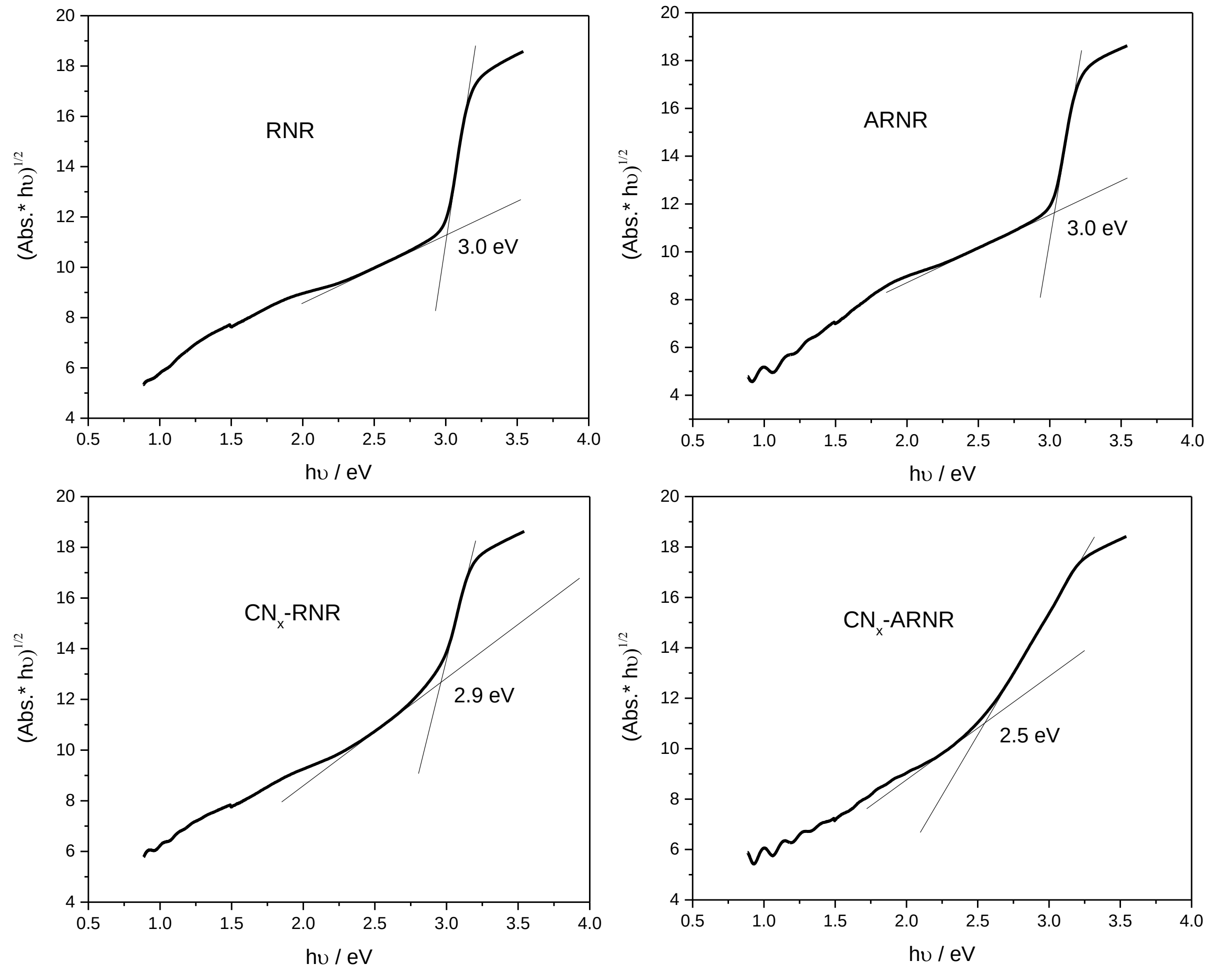

Figure S9 Tauc plots of RNR, ARNR, CN_x-RNR and CN_x-ARNR.

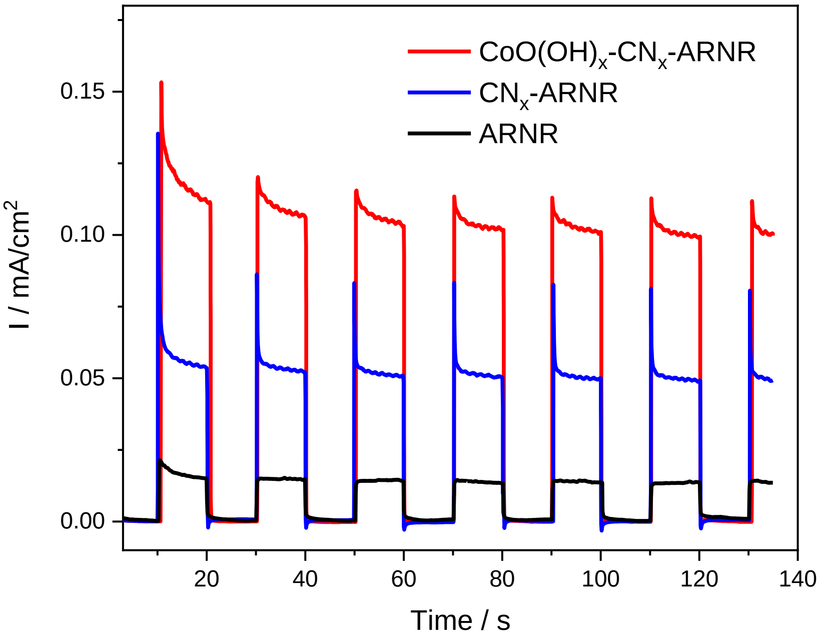

Figure S10 Chronoamperometric curves of ARNR-based electrodes (at applied potential of 0.98 *vs.* RHE in 0.1 M sodium borate buffer, pH=8).

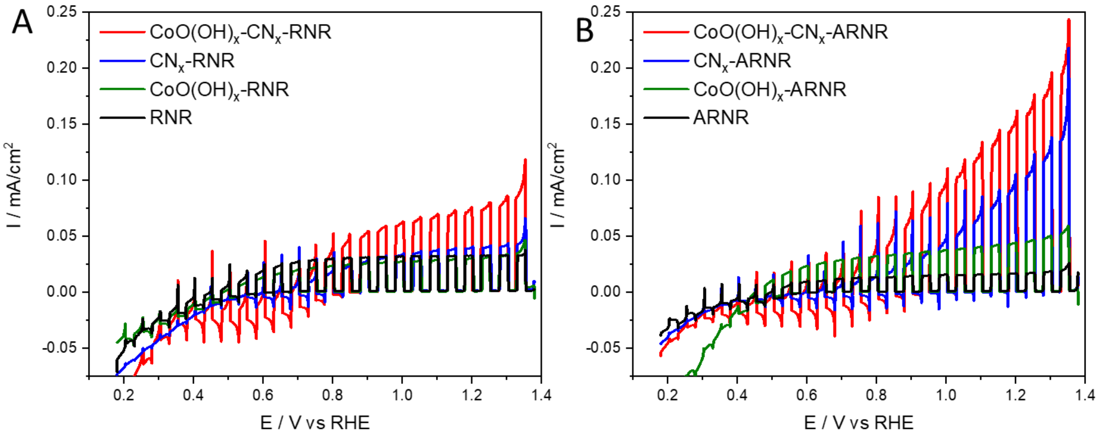

Figure S11 Photocurrent transients recorded in Na_2SO_4 (0.1 M, pH 8) under visible light irradiation λ >420 nm during cathodic potential sweep (5 mV/s) at RNR-based electrodes **(A)** and ARNR-based electrodes **(B)**.

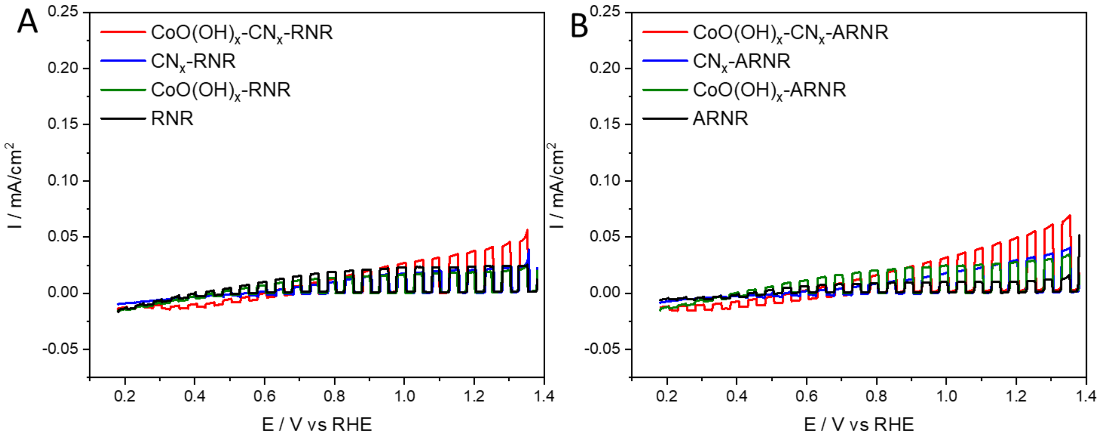

Figure S12 Photocurrent transients recorded in borate solution (0.1 M, pH 7) under visible light irradiation λ > 420 nm during cathodic potential sweep (5 mV/s) at RNR-based electrodes **(A)** and ARNR-based electrodes **(B)**.

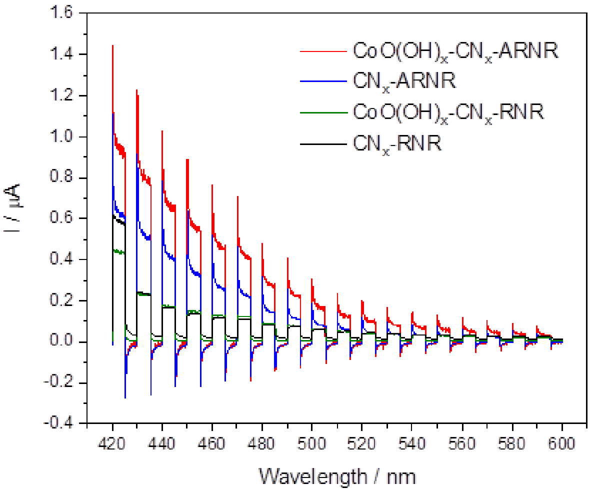

Figure S13 Transient photocurrent curves with IPCE measurement. Applied potential: 1.12 V *vs* RHE; electrolyte: 0.1 M borate solution (pH = 8).

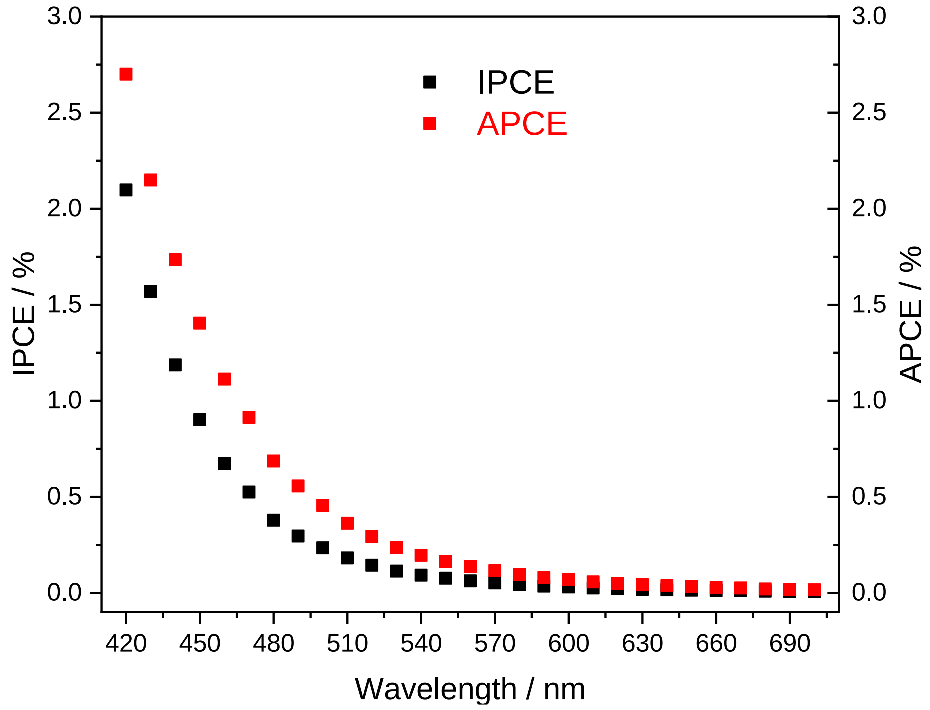

Figure S14 IPCE (incident photon-to-current efficiency) and APCE (absorbed photon-to-current efficiency) values recorded for of $CoO(OH)_x$-CN_x-ARNR electrode. Applied potential: 1.12 V *vs* RHE; electrolyte: 0.1 M borate solution (pH = 8).

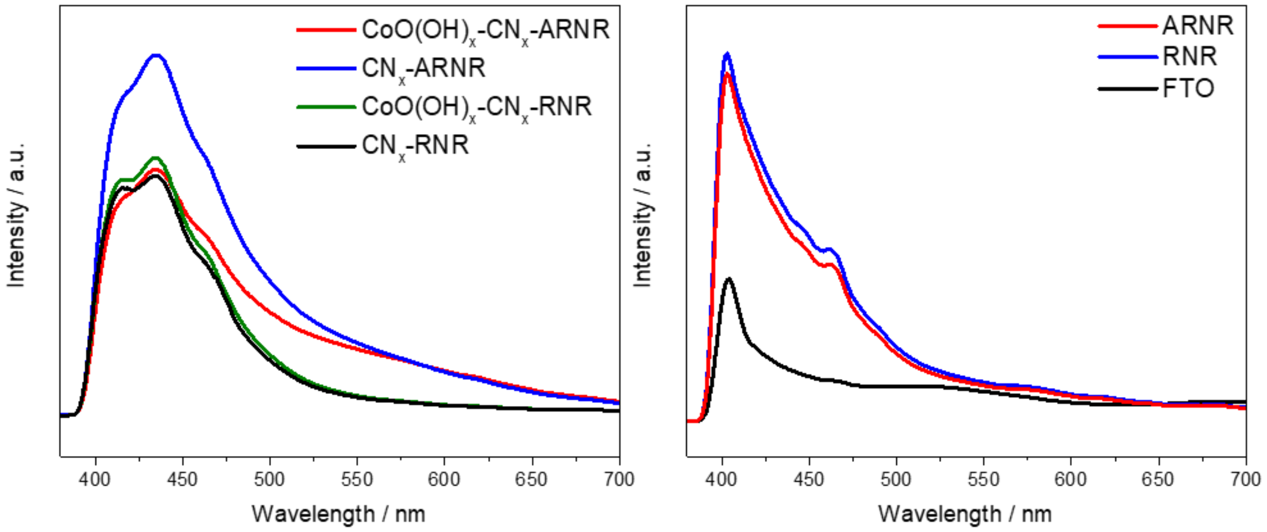

Figure S15 PL spectra: all CN_x-containing electrodes (left); ARNR, RNR and FTO (right).

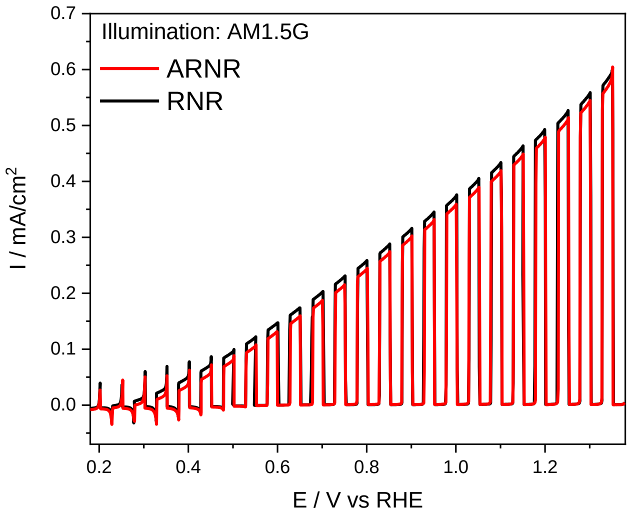

Figure S16 Photocurrents recorded for RNR and ARNR (*without* any CN_x or $CoO(OH)_x$) under full light of a solar simulator (1 sun). Electrolyte: 0.1 M borate solution (pH = 8). The negligible difference in photocurrent response indicates that there is no significant intrinsic effect of the rutile/anatase interface on the charge separation and transport.
